# Lactylation and viral infections: A novel link between metabolic reprogramming and immune regulation

**DOI:** 10.1371/journal.ppat.1013366

**Published:** 2025-07-28

**Authors:** Shanshan Chen, Tongxue Qin, Shengrui Luo, Fengyi Wang, Feirong Chen, Hailun Wei, Yuting Wu, Rongfeng Chen, Wudi Wei, Jingzhen Lai, Hao Liang, Li Ye, Zongxiang Yuan, Junjun Jiang

**Affiliations:** 1 Guangxi Key Laboratory of AIDS Prevention and Treatment & School of Public Health, Guangxi Medical University, Nanning, Guangxi, China; 2 Joint Laboratory for Emerging Infectious Diseases in China (Guangxi)-ASEAN, Life Sciences Institute, Guangxi Medical University, Nanning, Guangxi, China; Upstate Medical University, UNITED STATES OF AMERICA

## Abstract

Post-translational modifications (PTMs) regulate protein structure, function, and interactions, playing pivotal roles in cellular processes and disease progression. Lactate, a byproduct of the Warburg effect, accumulates excessively during viral infections and functions as a signaling molecule, disrupting mitochondrial antiviral-signaling protein activity and facilitating viral immune evasion. Lactylation, a recently identified PTM derived from lactate metabolism, links cellular metabolism and immune regulation by modulating gene expression and metabolic reprogramming. It also serves as a mechanism for viruses to modulate host immunity. Despite its emerging importance, its role with respect to viruses infecting humans and animals remains poorly understood. Investigating its impact on metabolic, protein modifications, and immune signaling may reveal novel immune evasion strategies and therapeutic targets. This review aims to provide an overview of the fundamental features and regulatory functions of lactylation, explore its association with viral infections, and offer insights into how lactylation influences metabolic and immune responses during virus–host interactions.

## Author summary

Viruses exploit host cellular machinery for replication, relying on host metabolic pathways to establish a conducive microenvironment for their proliferation. A notable consequence of this metabolic reprogramming is the accumulation of lactate, a glycolytic byproduct. Beyond its well-established role in metabolism, recent studies have identified lactate as a signaling molecule that drives lactylation, a newly discovered post-translational modification, that regulates gene expression, modulates immune responses, and influences cellular functionality. Despite these progresses, the role of lactylation in viral pathogenesis remains poorly characterized. In this review, we systematically examine the impact of lactylation on virus–host interactions, with particular emphasis on how viral pathogens exploit lactate-driven modifications to regulate host immune defenses, disrupt antiviral signaling cascades, and reprogram immune cell functionality. Lactylation modification establishes a critical link between cellular metabolism, epigenetic regulation, and immune modulation in the context of viral infections. By elucidating the mechanistic underpinnings of lactylation, we may reveal novel antiviral strategies and gain crucial insights into its regulatory roles in viral pathogenesis and host immunity.

## Introduction

Post-translational modifications (PTMs) of proteins are a class of important covalent modifications that occur after the translation of proteins to regulate their structure and function. As a key connection between gene expression and cellular functions, PTMs are important in regulating protein solubility, activity, stability, subcellular localization, and mediating protein interactions [1]. Common PTMs can facilitate virus–host interactions, including acetylation, glycosylation, methylation, phosphorylation, ADP-ribosylation, and ubiquitination [2]. Viruses cleverly use host protein PTMs to optimize their replication, assembly, and release, inhibit interferon (IFN) response, and promote viral proliferation and immune evasion [3]. Conversely, the host counters viral infection by neutralizing viral proteins, either by removing essential PTMs that are vital for their enzymatic activity or by attaching small molecules like ubiquitin or ubiquitin-like proteins, leading to their inactivation and/or proteasomal degradation, ultimately eliminating the infected cells [3]. Despite significant progress in recent years regarding the mechanisms of PTMs related to viral infections, many aspects of their specific regulatory networks remain unresolved.

Viruses are metabolically inert and rely on the host cell’s metabolism for replication and reproduction. To establish a favorable environment for the replication, they reprogram host metabolic pathways, such as glycolysis, the tricarboxylic acid cycle (TCA), and lipid metabolism. Among these altered metabolic pathways, glycolysis can provide rapid energy for viral replication and assembly but also induce a high accumulation of lactate in the host cell [4]. Lactate, a key carbon metabolite of the Warburg effect, not only regulates immune cell metabolism but also functions as a signaling molecule involved in the regulation of the immune response, including immunosurveillance and tumor cell escape mechanisms [5,6]. Furthermore, lactate is also the first metabolite identified to bind directly to mitochondrial antiviral-signaling protein (MAVS), which impairs its function and allows viruses to evade host defenses by inhibiting retinoic acid-inducible gene I (RIG-I) like receptors (RLR)-induced type-I interferons (IFNs) production [7].

Lactylation (Kla), a novel post-translational modification of lactate, has recently been identified as a key regulator of gene expression. First reported by Zhang and colleagues in 2019 that lactylation involves adding a lactyl (La) group to lysine residues in the tails of histone proteins [8]. Similar to other PTMs, lactylation plays a crucial role in regulating immune responses and maintaining biological homeostasis by converting cellular metabolic signals into transcriptional regulation, thus enabling cells to adapt to environmental changes [9]. For instance, lactylation promotes the polarization of macrophages toward an M2-like phenotype, suppressing immune responses within the tumor microenvironment (TME) [10]. Moreover, lactylation modulates metabolism-related gene expression, enhances cellular adaptability and plasticity, mediates immune cell reprogramming, and supports tissue repair following inflammation [11]. These findings highlight lactylation as a critical link between metabolic signaling and immune regulation.

Although recent studies, including a review [12], have begun to explore the role of lactylation in virology, comprehensive and systematic investigations on its functional mechanisms in virus–host interactions remain limited. Therefore, this review provides an overview of lactylation’s role in cellular metabolism and epigenetics, particularly focusing on its impact on immune responses in the field of viruses that infect humans and animals. We explore how lactylation affects host-viral interactions through metabolic reprogramming, protein modifications, and immune signaling pathways, highlighting key processes such as enhanced glycolysis and lactate accumulation. Additionally, we discuss the potential pathogenic role of lactylation and propose therapeutic strategies targeting lactylation in the context of viral infection. By establishing a theoretical framework, this study aims to deepen the understanding of the molecular interplay between lactylation and viral pathogenesis.

## Lactylation: a novel posttranslational modification

### Discovery of lactylation

**Fig 1:**
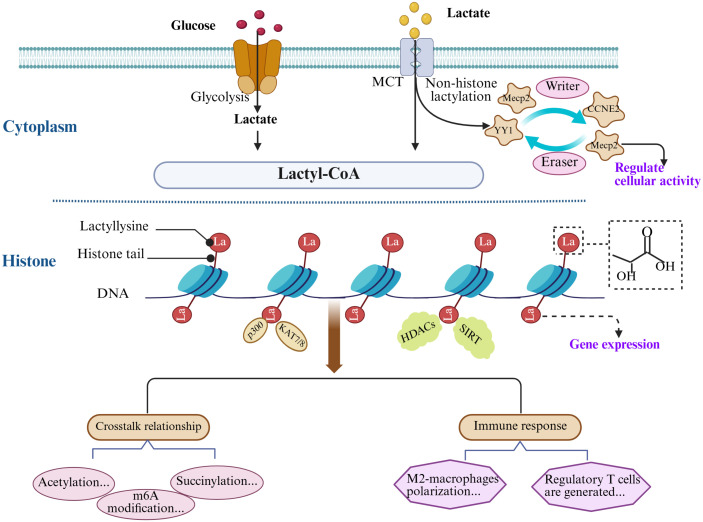
Lactate metabolism and the formation of lactylation.

In 2019, Zhang and colleagues employed mass spectrometry to detect a 72.021 Da mass shift on lysine residues of histones and proposed lactylation as an enzymatic PTM that uses lactyl coenzyme A (lactyl-CoA) as a substrate (Fig 1) [8]. Through isotope metabolic labeling, they demonstrated the widespread occurrence of lactylation in both human and mouse cells and identified 28 lactylation sites on the core histones [8]. They also further proved that lactylation levels correlated with lactate production, with both exogenous and endogenous lactate directly affecting this modification. Lactate that induces lactylation primarily exists in two forms: L-lactate (L-la) and D-lactate (D-la). L-la is predominantly produced through anaerobic glycolysis, particularly under hypoxic conditions, and its accumulation is markedly elevated in tumor cells due to the Warburg effect, reaching concentrations of 10–30 mM [13,14]. Elevated L-la contributes to an immunosuppressive microenvironment and promotes disease progression [15]. In contrast, D-la originates mainly from gut microbiota, diet, and the methylglyoxal pathway, and circulates at much lower levels (approximately 0.01 mM) [16,17]. It rises during conditions like intestinal injury and can impair mitochondrial respiration by disrupting the electron transport chain and increasing ROS [18,19].

Functionally, L-la serves as a substrate for enzymatic histone L-lactylation (K(L)-la), a modification primarily mediated by the histone acetyltransferase p300 and reversed by histone deacetylases HDAC1–3 [8,20]. K(L)-la plays an important role in linking glycolytic activity to transcriptional regulation. For instance, K(L)-la of cGAS suppresses cGAMP production, thereby modulating antiviral immune responses and tumor progression [21]. On the other hand, D-la promotes a distinct non-enzymatic lactylation pathway-histone D-lactylation (K(D)-la)-primarily through reaction with S-D-lactoylglutathione (LGSH), a byproduct of the glyoxalase pathway [22,23]. K(D)-la has been implicated in gene regulation; for example, it modifies histones to suppress TLR2 expression, thus inhibiting hepatocellular carcinoma (HCC) progression.

Despite their functional differences, detecting K(L)-la and K(D)-la poses substantial challenges due to their identical molecular weights and similar structural properties. Both forms are indistinguishable by high-performance liquid chromatography-mass spectrometry (HPLC-MS) [24], as peptides bearing K(L)-la and K(D)-la are not resolved by reverse-phase HPLC. Moreover, the much higher endogenous concentration of L-la relative to D-la leads to strong background signals, further complicating the detection of D-la-derived modifications [25].

Lactate, derived from glycolysis or the extracellular milieu or microenvironment, contributes to lactyl-CoA synthesis. Lactyl-CoA is a key intermediate in transferring lactyl groups to lysine residues on histones or non-histone proteins. Lactylation is catalyzed by “writers” enzymes such as p300 and KAT7/8, while “erasers” enzymes such as HDACs and SIRTs reverse the modification. Histone lactylation impacts gene expression, while non-histone lactylation influences cellular functions. BioRender supported the illustration rendering portion of this work (https://www.biorender.com/).

### The “writers” and “erasers” of lactylation modification

Like other epigenetic modifications, such as methylation and acetylation, lactylation modifications involve “writer” and “eraser” enzymes. The histone acetyltransferase p300, a crucial “writer”, induces histone H3 lysine acetylation and activates gene transcription by transferring the lactyl group from lactyl-CoA to protein lysine residues (Table 1). Zhang and colleagues have validated that p300 is involved in histone lactylation modification, as its overexpression in HEK293T cells increased histone lactylation levels, and its knockdown in both HCT116 and HEK293T cells decreased H3K18la levels [8]. Liu and colleagues further discovered that p300 mediates lactylation and regulates profibrotic gene expression by affecting lactylation levels [26]. In addition, CREB-binding protein (CBP), a homologous protein of p300, performs a similar function. Research by Li and colleagues showed that inhibiting the p300/CBP or blocking its enzyme activity with C646 attenuated lactate-induced lactylation levels in the high mobility group box 1 (HMGB1) immunocomplex [27]. YiaC, another “writer”, catalyzes lactyl-CoA formation and donates the lactyl group for lactylation, impacting metabolic enzyme activity [28]. As a critical lactyltransferase, KAT2A (also known as GCN5), in conjunction with ACSS2, forms a lactyltransferase complex that catalyzes histone lactylation at H3K14 and H3K18, which plays a critical role in tumor progression [29]. Additionally, KAT2A also catalyzes lysine lactylation at K124 of RCC2, and targeting this modification has been shown to suppress the rapid proliferation of breast cancer cells [30]. Beyond histone targets, emerging evidence suggests that KAT2A may also participate in the lactylation of cytoplasmic and mitochondrial proteins, although the mechanisms and biological consequences of these modifications remain to be fully elucidated. Recent research identified KAT8, a lysine acetyltransferase that acts as a pan-lactylation “writer”, enhances protein translation efficiency by lactylating of eEF1A2 at the K408 site, thereby promoting tumor progression [31]. Similarly, KAT7 is a “writing” enzyme involved in lactylation modification [32]. Moreover, AARS1 and AARS2 may act as “writers” and primarily function in the cytoplasm and mitochondria, respectively [33,34].

**Table 1:** The “writers” and “erasers” enzymes of lactylation modification.

Enzymes	Names	Roles	References
Writers	p300	Increase or decrease histone Kla levels in cells histone lactate and promotes its transcription Serve as a “writer” of lactylation on the YTHDF2 promoter in ocular melanoma cells	[8,38,39]
	p300/CBP	As a mediator of the most diverse lacyltransferase activities As important writers for HMGB1 lactylation in macrophages	[27,40]
	YiaC	Catalyze the addition of lysine lactylation both in vitro and intracellularly	[28]
	KAT2A	Catalyzes lactylation at histone sites H3K14 and H3K18 Mediates the RCC2 site 124 lysine lactylation	[29,30]
	KAT8	As a pan-Kla writer, KAT8 mediates Kla and serves as a molecular mechanism to promote tumor growth	[31]
	KAT7	Catalyzes a histone H3K9la and promotes the enzymatic activity for histone lactylation	[32]
	AARS1 and AARS2	Directly uses lactate and ATP to catalyze protein lactylation Mitochondrial AARS2 acts as a lysine lactyltransferase regulated by oxygen-sensing PHD2 via hydroxylation, triggering its degradation	[33,34]
Erasers	HDAC1–3	HDAC1–3 are highly efficient lysine delactylases, significantly decreasing global histone Kac and Kla levels in vitro Drastically reduce specific lactylation sites (H3K18la, H4K5la), with HDAC1 and HDAC3 exhibiting site-specific delactylase activity in cells	[20,35]
	HDAC6	HDAC6 acts as the primary lactyltransferase for α-tubulin, regulating microtubule dynamics	[41]
	SIRT1–3	SIRT1–3 robustly perform L-delactylation on overall histone lactylation as well as on H3K18(L-la) and H4K5(L-la)	[20]
	CobB	Increase PykF’s activity by erasing K382la, enhancing glycolysis, and bacterial growth	[28]

In contrast to “writers”, “erasers” terminate the lactylation cycle and maintain its dynamic balance by removing lactyl modifications from lysine residues. Histone deacetylases (HDACs), comprising HDAC1–11 and categorized into classes I, II, and IV, require Zn²⁺ as a cofactor for their activity. *In vitro* experiments have demonstrated that HDAC1 and HDAC3 reduce L-la and D-la levels by cleaving ε-N-L-lactyl lysine [35]. Zessin and colleagues further reported that HDAC6 and HDAC8 possess potential delactylase activity, although their enzymatic activity is considerably lower than that of HDAC3 [36]. Conversely, sirtuins (SIRT1–7), classified as class III HDACs, depend on NAD^+^ as a substrate [37]. Among the sirtuins, SIRT1–3 exhibited stronger activity in removing L-la, but HDAC3’s enzymatic activity in this process is several thousand times higher than SIRT2 (Table 1) [20]. Furthermore, Zhang and colleagues discovered CobB serves as the primary lysine delactylase regulating metabolism in *Escherichia coli*. It specifically modulates the activity of pyruvate kinase I (PykF) by regulating K382la, thereby promoting glycolysis and bacterial growth [28].

### Crosstalk between lactylation and other epigenomic events

It has been reported that many proteins contain at least one type of regulatory PTM [42]. Given that most proteins interact with other proteins to varying extents, the crosstalk between PTMs across different proteins is widespread. While research on the crosstalk between lactylation and other PTMs is still in its early stages, emerging studies indicate potential crosstalk between lactylation and acetylation [43,44]. Both commonly target lysine residues, and when occurring on histones, they often share sites such as H3K18, H3K27, and H3K23 [8]. Li and colleagues discovered that the Gli-like transcription factor 1 (Glis1) increases lactate and acetyl-CoA generation by regulating glycolytic gene expression, thereby enhancing the occurrence of lactylation and acetylation [44]. In CD4^+^ Th1 cells, glycolysis maintains acetyl-CoA levels during T-cell differentiation. Lactate dehydrogenase A (LDHA) is important in acetyl-CoA production, increasing histone acetylation at lysine 9 and 27 of H3 (H3K9Ac and H3K27Ac) and boosting IFN-γ expression in Th1 cells [45]. Moreover, p300 is the “writer” for lactylation and acetylation, providing further insights into their interrelationship. Research shows that p300 is highly enriched at the promoters of pluripotency genes (Oct4, Sall4, and c-Myc), suggesting that lactylation and acetylation may work synergistically to regulate cell fate through dynamic changes in lactate and acetyl-CoA levels (Fig 1) [46].

Besides acetylation, various other acylated modifications, including crotonylation, 2-hydroxyisobutyrylation, succinylation, and malonylation, may also co-modify target proteins alongside lysine lactylation, suggesting potential crosstalk among these modifications [47,48]. For example, Gao and colleagues identified a total of 143 sites on 83 lactylated proteins in B. cinerea that were commonly co-modified by lactylation, crotonylation and 2-hydroxyisobutyrylation, furtherly demonstrating the high conservation of acylated modifications among plant, human, and fungi [47]. Furthermore, in lipopolysaccharide (LPS)-activated macrophages, the succinylation of pyruvate kinase 2 at lysine K311 impairs glycolytic activity and drives its nuclear localization, promoting hypoxia-inducible factor (HIF)-dependent gene transcription and IL-1β production [49,50].

Additionally, significant crosstalk exists between lactylation and RNA modifications. Yu and colleagues have reported this potential connection, which between lactylation and RNA modifications, offering new perceptions of epigenetic regulation in carcinogenesis [51]. Specifically, lactate has been shown to induce methyltransferase-like 3 (METTL3) transcription in tumor-infiltrating myeloid cells (TIMs) via histone H3K18 lactylation, a marker associated with poor prognosis in colon cancer patients [52]. In addition to transcriptional regulation, Xiong and colleagues identified two lactylation sites within the zinc-finger domain (ZFD) of METTL3 that are critical for its RNA-binding ability, underscoring the role of lactylation in promoting METTL3-mediated m6A modifications, particularly in TIMs [53]. Notably, beyond its involvement in cancer, METTL3 also acts as a suppressor of antiviral immunity. Activators of the METTL3 RNA methyltransferase complex have been shown to enhance the production of viral particles in cells harboring the HIV-1 provirus [54]. During Vesicular Stomatitis Virus (VSV) infection, METTL3 translocates to the cytoplasm, promotes m6A modification of viral RNA, reduces viral dsRNA accumulation, and consequently attenuates RIG-I/MDA5-mediated antiviral signaling [55]. Given that lactylation upregulates METTL3 expression and enhances its RNA-binding capacity [52,53], it is plausible that lactylation may potentiate METTL3-driven immune suppression during viral infection. Although direct mechanistic evidence in viral contexts remains limited, this potential crosstalk may represent a novel strategy for viral immune evasion.

Beyond regulating METTL3 activity, lactylation also upregulates the expression of YTH N6-methyladenosine RNA binding protein 2 (YTHDF2), an m6A “reader” that selectively binds to m6A-modified RNAs, to the broader regulation of m6A-dependent processes. Mechanistically, lactylation increases YTHDF2 expression, enabling it to recognize m6A-modified Per1 and p53 mRNAs, promoting their degradation, thus accelerating the occurrence of ocular melanoma [51,56]. Although no literature has directly reported the involvement of lactylation in regulating YTHDF2 expression during viral infection, research has demonstrated that YTHDF2 binding to m6A sites on HIV-1 transcripts significantly enhances the stability of viral RNAs [57]. Furthermore, the downregulation of YTHDF2 appears to be linked to and potentially senses host shutoff induced by infection, thereby augmenting antiviral responses through the depression of interferon-stimulated gene (ISG) expression [58]. These findings highlight the importance of investigating whether lactylation modulates YTHDF2 expression or function during viral infection, which could reveal a novel layer of regulation in virus–host interactions.

### Lactylation mediates immune response

Excessive lactate contributes to the establishment of a microenvironment that supports cell growth and is crucial for shaping immune cell function [59]. Lactylation, a product of lactate metabolism, has been identified as a key regulatory mechanism in innate immune responses and a modulator of macrophage polarization. Macrophages exhibit two predominant phenotypes, pro-inflammatory (M1) and anti-inflammatory (M2), each playing specific roles in different pathophysiological processes [60–62]. Zhang and colleagues discovered that M1 macrophages possess an endogenous “lactate clock” that drives the late-stage transition to M2-type characteristics through lactylation (Fig 1) [8]. This modification increases in a time-dependent manner, promoting the overexpression of M2-like genes, such as arginine enzyme 1 (Arg-1), correlating positively with histone lactylation levels [8]. Further research by Noe and colleagues proposed that early M1 macrophages with high glycolytic/low TCA activity undergo lactylation, using lactate from glycolysis to initiate the M1 to M2 phenotypic transition in tumor-associated macrophages (TAMs). As glucose decreases and lactate levels increase in the microenvironment, M2 macrophages undergo metabolic reprogramming and exhibit higher TCA activity, which supports their anti-inflammatory phenotype and promotes tissue repair and tumor progression [63].

Beyond macrophage polarization, lactylation is also critical in regulating adaptive immune responses. Regulatory T (Treg) cells are well known for suppressing anti-tumor T cell activity, thereby maintaining an immunosuppressive microenvironment [64]. Research has shown that lactate promotes Treg cell generation by the lactylation of Lys72 in MOESIN (membrane-organizing extension spike protein), subsequently enhancing the TGF-β signaling pathway to upregulate the expression of forkhead box P3 (FOXP3). In contrast, the loss of FOXP3 results in immune dysregulation, contributing to tumorigenesis [65].

### The role of lactylation in viral infection

Viral infections trigger metabolic reprogramming in host cells, with enhanced glycolysis emerging as a hallmark feature. Elevated lactate levels, driven by glycolysis and LDH activity, suppress RLR-induced type-I IFN production in antiviral immunity, allowing pathogens to evade immune surveillance [7]. An intracellular surveillance protein is cyclic guanosine Monophosphate-Adenosine monophosphate (GMP-AMP) synthase (cGAS), a cytosolic DNA sensor. Lactylation at K21 of cGAS promotes tumor growth by accelerating cGAS protein degradation and suppressing interferon production through a ubiquitin-independent mechanism [66]. Emerging evidence further indicates that lactylation plays a significant role in viral infections, influencing the expression of viral and host genes, and modulating viral replication and host immune responses. In the following sections, we discuss the primary mechanisms that lactylation effects on viral infection and replication (Table 2).

**Table 2:** Roles and mechanisms of lactylation in various types of viruses.

Virus	Lactylation sites/proteins	Roles	Mechanisms	References
HBV	CENPA lactylation at site K124	Correlated with poor prognosis for HCC	Functioned as a transcriptional regulator to promote HCC via cooperating with YY1.	[67]
	H3K56la	Strengthen the tumorigenic ability of LCSCs	Enhanced the expression of the OCT4 gene.	[68]
	ALDOA lactylation at sites K230/322	Aggravated liver injury and HCC progression	Regulated ALDOA and DDX17 separation, promoted DDX17 entry into the nucleus, and aggravated HCC.	[68]
	SIRT3	Suppressed the development of HCC	Deacetylated non-histone proteins and prevented HCC development.	[69]
HPV16	G6PD lactylation at site K45	Promoted the rapid proliferation of tumor cells	HPV16 E6 promoted G6PD dimer formation by inhibiting its lactylation.	[70]
KSHV	NAT10 lactylation at site K290	Facilitated viral reactivation	The viral transcript PAN RNA facilitated the interaction between ATAT1 and NAT10, promoting the ac4C modification of tRNASer-CGA-1-1.	[71]
	ALKBH5	Strengthening the host’s antiviral immune response	Promoted the interaction between ALKBH5 and ESCO2 and reduced its binding to deacetylase SIRT6.	[72]
MPXV	ALKBH5	Strengthening the host’s antiviral immune response	Suppressed the expression of SIRT6 and impaired its recruitment by ALKBH5, thereby enhancing ALKBH5 lactylation.	[72]
HCMV	AARS1	Promotes viral spread	Induced lactylation of gluconeogenesis proteins, including pyruvate kinase M at lysine 62, also exhibits upregulated lactylation sites.	[73]
HSV-1	HIN-200 domain #2	Contributes to viral immune evasion	Impairing viral DNA-binding capacity.	[74]
SFTSV	YTHDF1 lactylation at sites K517/K521	Benefited from viral replication	SFTSV’s NSs protein binds with SIRT6, inhibiting the SIRT6-YTHDF1 complex formation.	[75]
PRRSV	H3K18la	Promoted viral growth	Activates the acetate-lactylation-HSPA6 axis to interfere with IFN-β induction.	[76]
WSSV	H3K18la and H4K12la	Boosted viral infection	Upregulated the expression of ribosomal protein S6 kinases 2 (S6K2) by H3K18la and H4K12la.	[4]

### Hepatitis B virus (HBV)

HBV is a double-stranded DNA virus with a small viral genome (3.2 kb), belonging to the Hepadnaviridae family. If left untreated, chronic HBV infection progresses to end-stage liver disease, such as liver cirrhosis and HCC [77]. Elevated serum lactate levels, recognized as a hallmark of liver dysfunction, have been confirmed as a simple and accurate prognostic marker [78]. Clinical research revealed that lactate levels are significantly higher in non-surviving patients with acute-on-chronic liver failure (ACLF) compared to survivors, and these also serve as a predictor of six-month mortality of patients with HBV-related decompensated cirrhosis [79]. Moreover, adjusting for lactate levels enhances the prognostic accuracy of established scoring systems like MELD and Child-Pugh scores [80].

**Fig 2:**
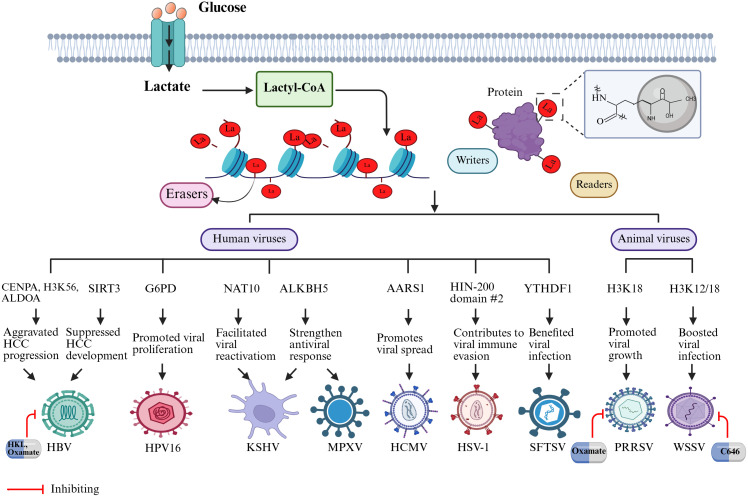
The mechanisms and therapeutic interventions of lactylation in human and animal viral pathogenesis.

As well as acting as a prognostic marker, lactate plays an active role in the pathogenesis of HBV by promoting lactylation, thereby facilitating liver injury and HCC progression. As an intracellular pathogen, HBV relies on host cell metabolism for replication, with the viral protein HBx being central in this process. Recent studies have shown that HBx reshapes the metabolic profile of infected hepatocytes by altering glycolysis and glycogen metabolism, inducing the Warburg effect by activating the mitogen-activated protein kinases (MAPK) signaling pathway. This metabolic reprogramming enhances HCC cell proliferation, promotes metastasis, and inhibits apoptosis [81,82]. Moreover, HBx enhances aerobic glycolysis by activating the NF-κBp65/hexokinase 2 (HK2) pathway, producing excessive lactate [83]. This accumulation of lactate further drives HCC progression through multiple mechanisms. It stimulates HCC cell proliferation via the PI3K/Akt signaling pathway to induce lactylation, while disrupting key signaling pathways such as Wnt/β-catenin, MAPK, and Notch, thereby exacerbating liver injury and driving tumor progression [83]. Building on these findings, Liao and colleagues identified centromere protein A (CENPA) as a vital transcriptional regulator in HCC, which lactylation at lysine 124 enables CENPA to cooperate with YY1 to upregulate target gene expression, enhancing its activation and accelerating tumorigenesis. Notably, high expression levels of CENPA correlate with poor prognosis (Fig 2) [67].

Liver cancer stem cells (LCSCs) are considered the primary drivers of phenotypic and functional heterogeneity in HCC and are associated with elevated levels of lactylation. As with HCC cells, LCSCs exhibit heightened glycolytic metabolism, increased lactate accumulation, and elevated lactylation levels. Importantly, this modification, exemplified by H3K56la, enhances OCT4 gene expression, specifically promoting tumorigenesis and influencing the proliferative capacity and stem cell properties of LCSCs [68]. Besides histone modifications, lactylation is critical in regulating non-histone proteins within LCSCs. For instance, lactylation at the K230 and K322 sites of aldolase A (ALDOA) diminishes its binding to the dead box deconjugate enzyme 17 (DDX17), facilitating the nuclear translocation of DDX17. Once in the nucleus, DDX17 drives stem cell-like properties of LCSCs and aggravates HCC [68]. Similarly, SIRT3 has been shown to regulate HCC progression through its impact on non-histone protein lactylation (Fig 2). Jin and colleagues proved that SIRT3 suppresses HCC development by delactylating cyclin E2 (CCNE2), a lactylated substrate of SIRT3, thereby reducing its ability to promote tumor growth in HCC cells [69].

These findings collectively emphasize the significant role of lactylation as a regulator of histone and non-histone proteins in HCC cells and LCSCs, targeting it may be a promising therapeutic avenue for improving outcomes in HBV-related liver disease and HCC.

### Human papillomaviruses (HPV)

HPV is a small, non-enveloped virus with a double-stranded circular DNA genome of 8 kb and encodes eight proteins, including the oncogenes E6 and E7. These oncogenes are essential for regulating cell cycle progression, inhibiting apoptosis, and evading immune response [84]. A hallmark of HPV infection is the reprogramming of host cell metabolism, which is driven by the E6 protein and facilitates rapid cell proliferation and viral replication [85]. Specifically, E6 promotes the degradation of p53, leads to increased glucose uptake through the overexpression of glucose transporter 1 (GLUT1), and further elevates glycolytic activity [86]. This metabolic shift not only promotes lactate accumulation but also alters cellular functions. Among its modulators is miR-34a, a tumor suppressor tightly regulated by p53. It exerts an anti-inflammatory effect by downregulating TNF-α and IL-6 [87] and suppresses lactate production by targeting LDHA [88]. However, HPV E6 degrades p53, resulting in decreased miR-34a levels and enhanced lactate production, ultimately fostering lactylation and potentially contributing to tumorigenesis [89]. These findings suggest that HPV-driven metabolic reprogramming enhances glycolysis and lactate production, potentially helping achieve an efficient viral replication.

The metabolic remodeling is also prominent in high-risk HPV types, such as HPV16, it accounts for more than half of cervical cancer cases [90]. Similar to other HPV types, HPV16 reprograms host metabolism by elevating glycolytic activity and lactate production, contributing to lactylation and tumor growth. In contrast to HPV E6, which promotes glycolysis by upregulating GLUT1, HPV16 oncoproteins E6 and E7 elevate lactate production by suppressing TP53-induced glycolysis and apoptosis regulator (TIGAR) expression through p53 degradation [91]. Subsequently, the accumulated lactate functions as a substrate for lactylation, impacting key cellular pathways related to tumor progression. Emerging evidence suggests that lactylation-modified proteins are predominantly enriched in glycolysis, the TCA cycle, and the pentose phosphate pathway (PPP), emphasizing the key role of lactylation in metabolic regulation [92]. The PPP is particularly noteworthy among these pathways, as it is often overactivated in tumor cells, supplying NADPH and metabolic intermediates that support cancer cell proliferation and tumor growth [93]. Notably, viral oncoproteins can directly influence PPP activity to promote tumor progression. For example, Meng and colleagues discovered that HPV16 E6 activates the PPP activity by inhibiting lactylation of glucose-6-phosphate dehydrogenase (G6PD) at K45, promoting its dimerization and increasing enzymatic activity (Fig 2). This activation of the PPP facilitates nucleotide synthesis, facilitating tumor cell proliferation [70]. Given the important role of lactylation in HPV16-driven metabolic reprogramming and tumor cell proliferation, developing new strategies to battle HPV16 infection by targeting lactylation is helpful.

### Herpesvirus and poxviruses

KSHV, also called Human Herpesvirus-8 (HHV-8), is a γ-2 herpesvirus with a 165–170 kb double-stranded linear DNA genome. It is the causative agent of diseases such as Kaposi’s sarcoma (KS) and primary effusion lymphoma (PEL) [94]. A characteristic of KSHV infection is the reprogramming of host cell metabolism, with enhanced glycolysis, increased lactate production, and reduced oxidative phosphorylation [95]. This metabolic transition drives the Warburg effect during latent infection and is important for tumor growth. In support of this, targeting glycolysis with specific inhibitors is a novel therapeutic approach for inhibiting latent KSHV infection [95]. Moreover, this metabolic vulnerability is also closely linked to the function of KSHV microRNAs within the oncogenic cluster, as they drive metabolic reprogramming by decreasing oxygen consumption, increasing lactate secretion and glucose uptake, and stabilizing HIF-1α. Mechanistically, these microRNAs downregulate hypoxia-inducible factor prolyl hydroxylase 2 (EGLN2) and mitochondrial heat shock protein A9 (HSPA9), leading to reduced mitochondrial copy number, which is a metabolic shift crucial for maintaining latency and providing a growth advantage [96].

In addition to KSHV microRNAs, the viral interferon regulatory factor 1 (vIRF1) promotes lactate secretion by activating the heterogeneous nuclear ribonuclear protein Q1 (hnRNP Q1) via the recruitment of the E3 ubiquitin ligase Kelch-like 3 (KLHL3). Through this interaction, hnRNP Q1 binds to the mRNA of glycerophosphodiester phosphodiesterase domain containing 1 (GDPD1), stabilizing it at the transcript level. However, this stabilization reduces GDPD1 protein expression, ultimately contributing to vIRF1-induced aerobic glycolysis [97]. Furthermore, the E3 ubiquitin ligase K5 further accelerates glucose consumption and lactate generation by directly altering receptor tyrosine kinase (RTK) endocytosis rates and downstream signaling [98]. This metabolic reprogramming in KSHV-infected cells drives elevated lactate levels and modulates lactylation processes, including NAT10 modification (Fig 2). As a lactylation “writer,” α-tubulin acetyltransferase 1 (ATAT1) interacts with NAT10 both *in vitro* and *in vivo*. This interaction is mediated by the viral transcript PAN RNA, enhancing NAT10 lactylation, promoting tRNASer-CGA-1-1 ac4C modification, and boosting KSHV lytic protein translation and viral reactivation [99].

Further to promoting viral replication, lactylation also shapes the host’s antiviral immune responses, particularly modulating key proteins like AlkB homolog 5 (ALKBH5). Li and colleagues found that lactylation of the m6A demethylase ALKBH5 is essential for establishing an effective immune response against KSHV (Fig 2). This lactylation modification is regulated by increased interactions with acetyltransferase ESCO2 and decreased interactions with deacetyltransferase SIRT6 during viral infections. Specifically, ALKBH5 binds to IFN-β mRNA, promoting the demethylation of its m6A modifications. Overexpression of ESCO2 or depletion of SIRT6 further enhances ALKBH5 lactylation, strengthening the host’s antiviral immune response [72]. This dual role of lactylation facilitates viral replication while improving the host immune system, emphasizing its pivotal role as a regulatory hub in the dynamic interplay between KSHV and host defense mechanisms.

Monkeypox virus (MPXV), a double-stranded DNA (dsDNA) virus, belongs to the Orthopoxvirus (OPXV) genus within the Poxviridae family [100]. Recurrent human monkeypox outbreaks in Africa, along with its accidental introduction into the United States in 2003, underscore the importance of maintaining vigilance against this orthopoxvirus as a potential emerging infectious disease [101]. Moreover, ecological disturbances and the emergence of co-infections such as HIV may further facilitate MPXV transmission [102]. Notably, the mechanism by which MPXV induces lactylation closely parallels that of Kaposi’s sarcoma-associated KSHV. In both cases, ALKBH5-mediated m6A RNA modification is regulated via lactylation, impacting type I interferon (IFN-I) responses. MPXV infection has also been shown to suppress the expression of SIRT6 and impair its recruitment by ALKBH5, thereby enhancing ALKBH5 lactylation and further modulating host antiviral defenses [72].

The other two herpesviruses-human cytomegalovirus (HCMV), a β-herpesvirus with a double-stranded linear DNA genome of approximately 230 kb, and herpes simplex virus 1 (HSV-1), a dsDNA virus with a complex structure that has evolved to replicate within human neurons and epithelial cells-are also capable of inducing significant metabolic reprogramming in host cells. This metabolic reprogramming is characterized by enhanced glycolysis and a Warburg-like effect, resulting in elevated lactate production and consequently increased protein lactylation [103]. However, the mechanisms and functional consequences of lactylation differ between these two viruses. During HCMV infection, lactylation facilitates viral spread, primarily mediated by the alanyl-tRNA synthetase (AlaRS) lactyltransferase activity of AARS1, which promotes to efficient cell-to-cell transmission. Mechanistically, HCMV infection induces lactylation of gluconeogenesis proteins, while canonical glycolysis proteins, including pyruvate kinase M (PKM) at lysine 62, also exhibit upregulated lactylation sites. In contrast, during HSV-1 infection of fibroblasts, neither exogenous lactate nor oxamate significantly alters viral titers, highlighting a fundamental difference from HCMV [73]. Despite these differences, both HCMV and HSV-1 target a common host restriction factor: interferon-γ-inducible protein 16 (IFI16), a nuclear viral DNA sensor that undergoes virus-induced lactylation, albeit at distinct sites [104]. In HCMV-infected cells, IFI16 lactylation is increased at multiple residues within the intrinsically disordered region (IDR) of IFI16, particularly at lysine 128 within the nuclear localization signal, a site previously known to be acetylated by p300 [105]. Conversely, HSV-1 increases lactylation primarily within the HIN-200 domain #2 of IFI16, potentially disrupting its DNA-binding ability. Notably, both infections induce conserved lactylation at lysine 90 in the IDR, a site not previously associated with acetylation [74]. Collectively, findings from Matthew and colleagues suggest that virus-induced lactate production may contribute to immune evasion by modulating lactylation of key host immune regulators, thereby impairing antiviral signaling pathways [73].

### Severe fever with thrombocytopenia syndrome virus (SFTSV)

SFTSV (Dabie bandavirus), a negative-stranded RNA virus from the Phenuiviridae family, causes severe clinical symptoms, including hemorrhagic fever and thrombocytopenia, leading to high fatality rates [106]. A clinical study found that the m6A demethylation gene FTO tended towards high expression after SFTSV infection, indicating the potential role of m6A modification in regulating viral replication and pathogenesis [107]. Beyond viral infections, m6A exerts profound effects in regulating RNA stability, localization, and translation, while also playing a pivotal role in tumor progression [108]. Recent research has shown that lactylation modulates the protein regulated by m6A. In ocular melanoma, elevated histone lactylation levels promote the expression of YTHDF2, binding to m6A-modified sites on PER1 and TP53 mRNAs, accelerating their degradation and facilitating tumor progression. Targeted correction of aberrant histone lactylation effectively inhibits tumorigenesis [51].

Similarly, m6A modification of SFTSV RNAs is crucial for regulating the virus’s infection and replication. Liu and colleagues found that SFTSV infection triggers m6A epigenetic modification in viral RNA, enabling recognition by the host’s YTHDF1 and leading to RNA degradation [75]. In response, the virus induces lactylation of YTHDF1 at the K517 and K521 sites, promoting its degradation and supporting viral replication (Fig 2). Mechanistically, ESCO1 and Sirt6 act as the “writer” and “eraser” of YTHDF1 lactylation, respectively, mutating the lactylation sites reduces YTHDF1 ubiquitination. Additionally, the virulence factor NSs of SFTSV competitively binds to Sirt6, inhibiting the formation of the Sirt6-YTHDF1 complex, which results in YTHDF1 degradation and counteracts the host’s degradation of m6A-modified viral RNAs [75]. Targeting viral-mediated lactylation of key m6A readers could enhance host defense.

### Porcine reproductive and respiratory syndrome virus (PRRSV)

PRRSV is an enveloped, positive-sense single-stranded RNA virus belonging to the Arteriviridae family. Since its discovery in 1987, PRRSV has become one of the most destructive pathogens in the global swine industry, causing significant economic losses [109]. Research has shown that PRRSV infection enhances the cellular uptake of glucose and glutamine, which are critical for viral replication. Depriving cells of either glucose or glutamine significantly reduces PRRSV replication [110]. It is worth noting that PRRSV relies on glycolysis and TCA cycle activity for its replication, while excessively elevated glycolysis or TCA cycle activity disrupts viral proliferation, suggesting that a finely metabolic environment is necessary for optimal viral replication [110]. This delicate balance is closely linked to key glycolytic enzymes that regulate energy metabolism. As an essential enzyme in glycolysis, glyceraldehyde-3-phosphate dehydrogenase (GAPDH) catalyzes the conversion of glyceraldehyde 3-phosphate to d-glycerate 1,3-bisphosphate, a crucial step in glycolysis. Inhibiting its activity could suppress aerobic glycolysis [111]. Beyond its metabolic role, GAPDH directly interacts with viral components during PRRSV infection. Liu and colleagues found that GAPDH binds to the PRRSV major envelope glycoprotein 5 (GP5), with the interaction occurring between 13-amino-acid (aa) region (aa 93–105) of GP5 and at Lys227 of GAPDH. This binding restricts GAPDH from translocating to the nucleus, redirecting it to the cytoplasm, where its glycolytic activity facilitates PRRSV replication [112].

In addition to glycolysis, PRRSV infection significantly enhances lactate production, a critical metabolite derived from glycolytic activity that directly promotes viral replication [113]. During PRRSV infection, elevated intracellular lactate levels induce lactylation of various proteins in both the cytoplasm and nucleus, thereby enabling the virus to manipulate host cell functions [110]. Pang and colleagues found that lactate is essential for optimal PRRSV proliferation and that its infection increases histone lactylation H3K18 (H3K18la) in a dose-dependent manner, underscoring its role as a key epigenetic mechanism facilitating viral immune evasion (Fig 2) [76]. Increased H3K18la directly activates HSPA6 expression, which inhibits the interaction between TNF receptor-associated factor 3 (TRAF3) and the IκB kinase subunit ε (IKKε), thus suppressing IFN-β production and weakening the host’s antiviral immune response [76]. This finding indicates that the lactate-lactylation-HSPA6 axis promotes viral growth by interfering with IFN-β induction, highlighting a potential therapeutic target for PRRSV infection.

### White spot syndrome virus (WSSV)

WSSV is a large, enveloped, rod-or elliptical-shaped dsDNA virus that mainly infects shrimp, causing severe damage to the shrimp aquaculture industry [114]. During the viral genome replication stage (12 h postinfection), WSSV infection activates aerobic glycolysis, glutamine metabolism, and the PPP, which collectively increase glucose consumption and plasma lactate levels, playing a crucial role in supporting viral replication and pathogenesis [115]. This metabolic reprogramming is essential for viral replication and is linked to manipulating host enzymes to sustain infection. In this context, viral components, such as palmitic amide packaged in WSSV virions, bind to triosephosphate isomerase (TPI), enhancing its enzymatic activity, promoting glycolysis, and playing a pivotal role in initiating the viral life cycle within the host cytoplasm. This interaction leads to lactate accumulation, which upregulates HIF-1 expression and intensifies glycolysis, establishing a positive feedback loop that facilitates viral infection [116].

Beyond driving metabolic reprogramming, lactate regulates histone lactylation within host cells, creating an epigenetic environment to support viral replication. This shift in cellular metabolism is reflected in the alteration of key enzymes and histone modifications. Zhang and colleagues found that WSSV infection significantly increased the levels of key glycolytic enzymes, such as HK and LDH, in hemocytes and intestinal tissues of the infected shrimp, enhancing glycolysis and lactate accumulation, and increasing the lactylation levels of H3K18 and H4K12 [4]. In this context, shrimp p300 acted as the “writer” of lactylation, while HDAC1 and HDAC3 functioned as lysine delactylases to regulate lactylation levels. Further analysis revealed that H3K18la and H4K12la modifications were enriched in the promoters of 75 target genes, including ribosomal protein S6 kinases 2 (S6K2) (Fig 2). This upregulation of S6K2 promotes viral infection. Interestingly, the virus-encoded miR-N20 targets HIF-1α, inhibiting glycolysis and suppressing H3K18la and H4K12la modification [4]. This finding highlights the pivotal role of lactylation in WSSV infection, driving metabolic reprogramming for viral replication and maintaining lactylation levels to sustain infection persistence.

In summary, lactate-derived lactylation influences the replication and transmission of various viruses through histone and non-histone lactylation sites across multiple pathways (Fig 2).

Histone and/or non-histone lactylation regulate the proliferation and infection of human and animal viruses by driving the expression of various genes. The figure highlights potential therapeutic strategies targeting lactylation pathways in different viruses, such as HKL, oxamate, and C646, underscoring their role in inhibiting viral replication and modulating host metabolism. BioRender supported the illustration rendering portion of this work (https://www.biorender.com/).

### Therapeutic potential of targeting lactylation in virus infection

The lactylation process involves lactate generation, transport, and regulation by “writers” or “erasers” all representing potential therapeutic targets [117]. To fully utilize this potential, a deeper understanding of lactylation mechanisms and their specific targets in viral infections is crucial, paving the way for developing novel treatment strategies.

### Modulation of enzymes involved in lactylation

One key approach to targeting lactylation involves modulating its “writers” and “erasers”, the enzymes that regulate the addition and removal of lactylation marks. Lactyltransferases, as the “writers”, play a central role in this modification. Similarly, “erasers” like SIRT enzymes counterbalance this process by removing lactylation. These processes regulate intracellular lactylation levels by targeting specific enzymes, providing novel therapeutic opportunities. For instance, the p300/CBP inhibitor, C646 effectively suppresses WSSV replication in infection models by reducing H3K18la and H4K12la lactylation levels (Fig 2) [4]. Additionally, SIRT3, a key delactylase that targets CCNE2, has been identified as a potential therapeutic target for liver cancer treatment. Honokiol (HKL), a SIRT3 activator, directly enhances delactylation enzyme activity, effectively inhibiting HCC growth in *vivo* (Fig 2) [69]. This emphasizes the therapeutic potential of targeting delactylation pathways in disease treatment. Notably, lactylation shares “writers” and “erasers” with other PTMs, particularly acetylation, which increases the risk of off-target effects, highlighting the need for further investigation into drugs targeting p300 and SIRT regulation.

### Modulating metabolic pathways associated with lactylation modification

Targeting the metabolism of virus-infected cells, particularly by inhibiting glycolysis or modulating other metabolic pathways, may offer an indirect strategy to reduce lactylation. For example, Demethylzeylasteral (DML), a triterpene antitumor compound, has been shown to effectively suppress the tumorigenesis of LCSCs by reducing lactate levels and inhibiting H3 histone lactylation [118]. Building on these findings, further metabolic interventions could focus on targeting specific enzymes and transporters that are critical to glycolysis and lactate metabolism, thereby modulating lactylation and its associated cellular processes.

LDH is a key enzyme in glycolysis that catalyzes the conversion of pyruvate to lactate while regenerating NAD^+^ from NADH, a critical step for sustaining glycolysis and ATP production [119]. Since lactate is the direct substrate for lactylation, inhibiting LDH reduces intracellular lactate concentrations and lowers lactylation levels. In WSSV-infected shrimp, silencing LDH significantly reduces viral loads and mortality [4]. Similarly, Oxamate, an LDH inhibitor, reduces lactate production and lactylation, impairing cellular glycolysis and restricting viral replication such as HBV, PRRSV, and SVA (Fig 2) [113,120–122]. Beyond intracellular lactate metabolism, extracellular lactate transport also plays a vital role in shaping the metabolic microenvironment. Monocarboxylate transporters (MCTs), primarily MCT1 and MCT4, regulate lactate flux across the plasma membrane, with MCT1 facilitating lactate import and MCT4 driving its export [123]. Although the role of MCT inhibitors in viral infections is unreported, targeting MCT1 has been shown to enhance anti-tumor immune cell infiltration, such as dendritic and natural killer cells, suppressing tumor growth. For example, the MCT1 inhibitor AZD3965 reduces lactate export, reprograms metabolism, and promotes immune cell activity to inhibit tumor progression [124]. Furthermore, virus-specific MCT1-deficient CD8^+^ T cells fail to establish memory T cells, which are crucial for controlling γ-herpesvirus reactivation during latency. This highlights that lactate export is essential for generating protective memory T cells needed to prevent viral latency from progressing into chronic infection [125]. Although significant progress has been made in utilizing lactylation to address viral infection, further research is essential to uncover its underlying mechanisms.

## Conclusion and perspectives

The study of lactylation as a novel PTM is essential for understanding its functional and regulatory mechanisms in physiological and pathological processes, providing valuable insights into disease development. Lactylation has been implicated in diverse processes, including cellular development and differentiation, inflammation, tumor progression, and neurological disorders [126,127]. Moreover, emerging evidence links lactylation to the replication and spread of viruses [4,99]. This review systematically examines the molecular mechanisms and regulatory pathways of lactylation, with a particular focus on its role in viral pathogenesis. We highlight the therapeutic potential of targeting lactylation in managing infectious diseases, considering its impact on infection progression and immune modulation.

To elucidate the role of lactylation in viral infections, we examined its functions throughout the viral life cycle and its impact on host cellular processes. Lactylation facilitates viral replication by reprogramming host metabolism, particularly through the upregulation of glycolysis, providing the energy and biosynthetic intermediates required for viral proliferation [76]. The resulting lactate accumulation serves as a substrate for lactylation, driving histone modifications such as H3K18la and H4K12la, which promote the expression of viral and host genes crucial for infection [4]. Simultaneously, lactylation of non-histone proteins disrupts immune signaling pathways, such as cGAS and RLR, which interferes with the IFN responses and ultimately suppresses the host’s antiviral defense [7,66]. These findings emphasize lactylation’s dual role in viral infections: facilitating viral replication through metabolic reprogramming and gene expression while aiding immune evasion by impairing antiviral pathways, indicating it could be a promising therapeutic target for antiviral strategies.

Although significant progress has been made, research on lactylation induced by viral infection is still in its early stages, and several key aspects require further exploration. First, the regulatory mechanisms of lactylation during virus infection remain unclear. While lactylation significantly regulates metabolism, gene expression, and immune responses, these functions are not exclusive to viral infections. Further research must elucidate its precise molecular roles, particularly the mechanisms involved in non-histone protein lactylation. Second, viral infections often involve multiple PTMs, such as glycosylation, phosphorylation, acylation, and methylation [128,129]. Lactylation may interact with these modifications through synergistic or antagonistic mechanisms, emphasizing the need to explore the complex interplay and collective impact on viral pathogenesis. Third, while targeting lactylation holds therapeutic potential for infectious diseases, it also presents challenges. Developing drugs to modulate lactylation requires careful study to ensure efficacy and safety, minimizing toxicity to normal cells and avoiding essential physiological processes disruption. Combining lactylation regulation with immunotherapy has shown promise for enhancing therapeutic outcomes [130–132], but further clinical trials are needed to validate its feasibility and effectiveness. Additionally, challenges like treatment durability, resistance development, and potential side effects must be addressed to pave the way for successful clinical applications.

In summary, our understanding of lactylation in viral infection is still evolving. Further research is crucial to identify safe targets among lactylation regulators that could mitigate diseases caused by viruses and facilitate the development of effective treatments. Future clinical research should optimize these approaches to enhance treatment efficacy and broaden their applicability.

## Abbreviations

ACLF: acute-on-chronic liver failure
CENPA: centromere protein A
HBV: hepatitis B virus
HCC: hepatocellular carcinoma
IDR: intrinsically disordered region
IFN: interferon
ISG: interferon-stimulated gene
LCSCs: liver cancer stem cells
MAVS: mitochondrial antiviral-signaling protein
PPP: pentose phosphate pathway
PTMs: post-translational modifications
RTK: receptor tyrosine kinase
TAMs: tumor-associated macrophages
TME: tumor microenvironment

## References

[1] Uy R, Wold F. Posttranslational covalent modification of proteins. Science. 1977;198(4320):890–6. doi: 10.1126/science.337487 337487

[2] Cheng N, Liu M, Li W, Sun B, Liu D, Wang G, et al. Protein post-translational modification in SARS-CoV-2 and host interaction. Front Immunol. 2023;13:1068449. doi: 10.3389/fimmu.2022.1068449 36713387 PMC9880545

[3] Kumar R, Mehta D, Mishra N, Nayak D, Sunil S. Role of host-mediated post-translational modifications (PTMs) in RNA virus pathogenesis. Int J Mol Sci. 2020;22(1). 10.3390/ijms22010323 PMC7796338 33396899

[4] Zhang Y, Zhang X. Virus-induced histone lactylation promotes virus infection in crustacean. Adv Sci (Weinh). 2024;11(30):e2401017. doi: 10.1002/advs.202401017 38874057 PMC11321649

[5] Pucino V, Cucchi D, Mauro C. Lactate transporters as therapeutic targets in cancer and inflammatory diseases. Expert Opin Ther Targets. 2018;22(9):735–43. doi: 10.1080/14728222.2018.1511706 30106309

[6] Böttcher M, Baur R, Stoll A, Mackensen A, Mougiakakos D. Linking immunoevasion and metabolic reprogramming in B-cell-derived lymphomas. Front Oncol. 2020;10:594782. doi: 10.3389/fonc.2020.594782 33251150 PMC7674840

[7] Zhang W, Wang G, Xu Z-G, Tu H, Hu F, Dai J, et al. Lactate is a natural suppressor of RLR signaling by targeting MAVS. Cell. 2019;178(1):176-189.e15. doi: 10.1016/j.cell.2019.05.003 31155231 PMC6625351

[8] Zhang D, Tang Z, Huang H, Zhou G, Cui C, Weng Y, et al. Metabolic regulation of gene expression by histone lactylation. Nature. 2019;574(7779):575–80. doi: 10.1038/s41586-019-1678-1 31645732 PMC6818755

[9] Wang X, Ying T, Yuan J, Wang Y, Su X, Chen S, et al. BRAFV600E restructures cellular lactylation to promote anaplastic thyroid cancer proliferation. Endocr Relat Cancer. 2023;30(8):e220344. doi: 10.1530/ERC-22-0344 37184950

[10] Chen L, Huang L, Gu Y, Cang W, Sun P, Xiang Y. Lactate-lactylation hands between metabolic reprogramming and immunosuppression. Int J Mol Sci. 2022;23(19). 10.3390/ijms231911943 PMC9569569 36233246

[11] Yu X, Yang J, Xu J, Pan H, Wang W, Yu X, et al. Histone lactylation: from tumor lactate metabolism to epigenetic regulation. Int J Biol Sci. 2024;20(5):1833–54. doi: 10.7150/ijbs.91492 38481814 PMC10929197

[12] Wang S, Zhang L. The role of lactylation in virology. Virology. 2025;605:110466. doi: 10.1016/j.virol.2025.110466 40020541

[13] Adeva-Andany M, López-Ojén M, Funcasta-Calderón R, Ameneiros-Rodríguez E, Donapetry-García C, Vila-Altesor M, et al. Comprehensive review on lactate metabolism in human health. Mitochondrion. 2014;17:76–100. doi: 10.1016/j.mito.2014.05.007 24929216

[14] Li J, Ma P, Liu Z, Xie J. L- and D-lactate: unveiling their hidden functions in disease and health. Cell Commun Signal. 2025;23(1):134. doi: 10.1186/s12964-025-02132-z 40075490 PMC11905701

[15] Faubert B, Li KY, Cai L, Hensley CT, Kim J, Zacharias LG, et al. Lactate metabolism in human lung tumors. Cell. 2017;171(2):358-371.e9. doi: 10.1016/j.cell.2017.09.019 28985563 PMC5684706

[16] Levitt MD, Levitt DG. Quantitative evaluation of D-lactate pathophysiology: new insights into the mechanisms involved and the many areas in need of further investigation. Clin Exp Gastroenterol. 2020;13:321–37. doi: 10.2147/CEG.S260600 32982363 PMC7490090

[17] Jin S, Chen X, Yang J, Ding J. Lactate dehydrogenase D is a general dehydrogenase for D-2-hydroxyacids and is associated with D-lactic acidosis. Nat Commun. 2023;14(1):6638. doi: 10.1038/s41467-023-42456-3 37863926 PMC10589216

[18] Forsyth RJ, Moulden A, Hull D. D-lactate associated encephalopathy in short bowel syndrome: management with long-term non-absorbable oral antimicrobials. Clin Nutr. 1991;10(6):352–5. 16839944 10.1016/0261-5614(91)90066-l

[19] Quiroga J, Alarcón P, Ramírez MF, Manosalva C, Teuber S, Carretta MD, et al. D-lactate-induced ETosis in cattle polymorphonuclear leucocytes is dependent on the release of mitochondrial reactive oxygen species and the PI3K/Akt/HIF-1 and GSK-3β pathways. Dev Comp Immunol. 2023;145:104728. doi: 10.1016/j.dci.2023.104728 37164278

[20] Moreno-Yruela C, Zhang D, Wei W, Bæk M, Liu W, Gao J, et al. Class I histone deacetylases (HDAC1-3) are histone lysine delactylases. Sci Adv. 2022;8(3):eabi6696. doi: 10.1126/sciadv.abi6696 35044827 PMC8769552

[21] Li H, Liu C, Li R, Zhou L, Ran Y, Yang Q, et al. AARS1 and AARS2 sense L-lactate to regulate cGAS as global lysine lactyltransferases. Nature.2024;634(8036),1229–37. 39322678 10.1038/s41586-024-07992-y

[22] Rabbani N, Xue M, Thornalley PJ. Activity, regulation, copy number and function in the glyoxalase system. Biochem Soc Trans. 2014;42(2):419–24. 24646254 10.1042/BST20140008

[23] Gaffney DO, Jennings EQ, Anderson CC, Marentette JO, Shi T, Schou Oxvig A-M, et al. Non-enzymatic lysine lactoylation of glycolytic enzymes. Cell Chem Biol. 2020;27(2):206-213.e6. doi: 10.1016/j.chembiol.2019.11.005 31767537 PMC7395678

[24] Zhang D, Gao J, Zhu Z, Mao Q, Xu Z, Singh PK, et al. Lysine L-lactylation is the dominant lactylation isomer induced by glycolysis. Nat Chem Biol. 2025;21(1):91–9. doi: 10.1038/s41589-024-01680-8 39030363 PMC11666458

[25] Gao J, Liu R, Huang K, Li Z, Sheng X, Chakraborty K, et al. Dynamic investigation of hypoxia-induced L-lactylation. Proc Natl Acad Sci U S A. 2025;122(10):e2404899122. doi: 10.1073/pnas.2404899122 40030031 PMC11912421

[26] Cui H, Xie N, Banerjee S, Ge J, Jiang D, Dey T, et al. Lung myofibroblasts promote macrophage profibrotic activity through lactate-induced histone lactylation. Am J Respir Cell Mol Biol. 2021;64(1):115–25. doi: 10.1165/rcmb.2020-0360OC 33074715 PMC7780997

[27] Yang K, Fan M, Wang X, Xu J, Wang Y, Tu F, et al. Lactate promotes macrophage HMGB1 lactylation, acetylation, and exosomal release in polymicrobial sepsis. Cell Death Differ. 2022;29(1):133–46. doi: 10.1038/s41418-021-00841-9 34363018 PMC8738735

[28] Dong H, Zhang J, Zhang H, Han Y, Lu C, Chen C, et al. YiaC and CobB regulate lysine lactylation in Escherichia coli. Nat Commun.2022;13(1),6628. 36333310 10.1038/s41467-022-34399-y PMC9636275

[29] Zhu R, Ye X, Lu X, Xiao L, Yuan M, Zhao H, et al. ACSS2 acts as a lactyl-CoA synthetase and couples KAT2A to function as a lactyltransferase for histone lactylation and tumor immune evasion. Cell Metab. 2025;37(2):361-376.e7. doi: 10.1016/j.cmet.2024.10.015 39561764

[30] Zheng B, Pan Y, Qian F, Liu D, Ye D, Yu B, et al. High sugar induced RCC2 lactylation drives breast cancer tumorigenicity through upregulating MAD2L1. Adv Sci (Weinh). 2025;12(21):e2415530. doi: 10.1002/advs.202415530 40145796 PMC12140329

[31] Xie B, Zhang M, Li J, Cui J, Zhang P, Liu F, et al. KAT8-catalyzed lactylation promotes eEF1A2-mediated protein synthesis and colorectal carcinogenesis. Proc Natl Acad Sci U S A. 2024;121(8):e2314128121. doi: 10.1073/pnas.2314128121 38359291 PMC10895275

[32] Niu Z, Chen C, Wang S, Lu C, Wu Z, Wang A, et al. HBO1 catalyzes lysine lactylation and mediates histone H3K9la to regulate gene transcription. Nat Commun. 2024;15(1):3561. doi: 10.1038/s41467-024-47900-6 38670996 PMC11053077

[33] Mao Y, Zhang J, Zhou Q, He X, Zheng Z, Wei Y, et al. Hypoxia induces mitochondrial protein lactylation to limit oxidative phosphorylation. Cell Res. 2024;34(1):13–30. doi: 10.1038/s41422-023-00864-6 38163844 PMC10770133

[34] Ju J, Zhang H, Lin M, Yan Z, An L, Cao Z. The alanyl-tRNA synthetase AARS1 moonlights as a lactyltransferase to promote YAP signaling in gastric cancer. J Clin Invest. 2024;134(10). 10.1172/JCI174587 PMC11093599 38512451

[35] Audia JE, Campbell RM. Histone modifications and cancer. Cold Spring Harb Perspect Biol. 2016;8(4):a019521. doi: 10.1101/cshperspect.a019521 27037415 PMC4817802

[36] Zessin M, Meleshin M, Praetorius L, Sippl W, Bařinka C, Schutkowski M. Uncovering robust delactoylase and depyruvoylase activities of HDAC isoforms. ACS Chem Biol. 2022;17(6):1364–75. doi: 10.1021/acschembio.1c00863 35639992

[37] Hong YA, Kim JE, Jo M, Ko GJ. The role of sirtuins in kidney diseases. Int J Mol Sci. 2020;21(18). 10.3390/ijms21186686 PMC7555196 32932720

[38] Li X, Yang Y, Zhang B, Lin X, Fu X, An Y, et al. Lactate metabolism in human health and disease. Signal Transduct Target Ther. 2022;7(1):305. doi: 10.1038/s41392-022-01151-3 36050306 PMC9434547

[39] Varner EL, Trefely S, Bartee D, von Krusenstiern E, Izzo L, Bekeova C, et al. Quantification of lactoyl-CoA (lactyl-CoA) by liquid chromatography mass spectrometry in mammalian cells and tissues. Open Biol. 2020;10(9):200187. doi: 10.1098/rsob.200187 32961073 PMC7536085

[40] Dancy BM, Cole PA. Protein lysine acetylation by p300/CBP. Chem Rev. 2015;115(6):2419–52. doi: 10.1021/cr500452k 25594381 PMC4378506

[41] Sun S, Xu Z, He L, Shen Y, Yan Y, Lv X, et al. Metabolic regulation of cytoskeleton functions by HDAC6-catalyzed α-tubulin lactylation. Nat Commun. 2024;15(1):8377. doi: 10.1038/s41467-024-52729-0 39333081 PMC11437170

[42] Ji F, Zhou M, Zhu H, Jiang Z, Li Q, Ouyang X. Integrative proteomic analysis of multiple posttranslational modifications in inflammatory response. Genomics Proteomics Bioinformatics. 2022;20(1):163–76. 33662623 10.1016/j.gpb.2020.11.004 PMC9510875

[43] Notarangelo G, Haigis MC. Sweet temptation: from sugar metabolism to gene regulation. Immunity. 2019;51(6):980–1. doi: 10.1016/j.immuni.2019.11.008 31851904

[44] Li L, Chen K, Wang T, Wu Y, Xing G, Chen M, et al. Glis1 facilitates induction of pluripotency via an epigenome-metabolome-epigenome signalling cascade. Nat Metab. 2020;2(9):882–92. doi: 10.1038/s42255-020-0267-9 32839595

[45] Peng M, Yin N, Chhangawala S, Xu K, Leslie CS, Li MO. Aerobic glycolysis promotes T helper 1 cell differentiation through an epigenetic mechanism. Science. 2016;354(6311):481–4. doi: 10.1126/science.aaf6284 27708054 PMC5539971

[46] Dai X, Lv X, Thompson EW, Ostrikov KK. Histone lactylation: epigenetic mark of glycolytic switch. Trends Genet. 2022;38(2):124–7. doi: 10.1016/j.tig.2021.09.009 34627643

[47] Gao M, Zhang N, Liang W. Systematic analysis of lysine lactylation in the plant fungal pathogen Botrytis cinerea. Frontiers in Microbiology. 2020;11:594743. 33193272 10.3389/fmicb.2020.594743 PMC7649125

[48] Meng X, Baine JM, Yan T, Wang S. Comprehensive analysis of lysine lactylation in rice (*Oryza sativa*) grains. J Agric Food Chem. 2021;69(29):8287–97. doi: 10.1021/acs.jafc.1c00760 34264677

[49] Wang F, Wang K, Xu W, Zhao S, Ye D, Wang Y, et al. SIRT5 desuccinylates and activates pyruvate kinase M2 to block macrophage IL-1β production and to prevent DSS-induced colitis in mice. Cell Rep. 2017;19(11):2331–44. doi: 10.1016/j.celrep.2017.05.065 28614718

[50] Palsson-McDermott EM, Curtis AM, Goel G, Lauterbach MAR, Sheedy FJ, Gleeson LE, et al. Pyruvate kinase M2 regulates Hif-1α activity and IL-1β induction and is a critical determinant of the warburg effect in LPS-activated macrophages. Cell Metab. 2015;21(2):347. doi: 10.1016/j.cmet.2015.01.017 29510100

[51] Yu J, Chai P, Xie M, Ge S, Ruan J, Fan X, et al. Histone lactylation drives oncogenesis by facilitating m6A reader protein YTHDF2 expression in ocular melanoma. Genome Biol. 2021;22(1):85. doi: 10.1186/s13059-021-02308-z 33726814 PMC7962360

[52] Lan H, Liu Y, Liu J, Wang X, Guan Z, Du J. Tumor-associated macrophages promote oxaliplatin resistance via METTL3-mediated m(6)A of TRAF5 and necroptosis in colorectal cancer. Mol Pharm. 2021;18(3):1026–37. 33555197 10.1021/acs.molpharmaceut.0c00961

[53] Xiong J, He J, Zhu J, Pan J, Liao W, Ye H, et al. Lactylation-driven METTL3-mediated RNA m6A modification promotes immunosuppression of tumor-infiltrating myeloid cells. Mol Cell. 2022;82(9):1660-1677.e10. doi: 10.1016/j.molcel.2022.02.033 35320754

[54] Selberg S, Žusinaite E, Herodes K, Seli N, Kankuri E, Merits A, et al. HIV replication is increased by RNA methylation METTL3/METTL14/WTAP complex activators. ACS Omega. 2021;6(24):15957–63. doi: 10.1021/acsomega.1c01626 34179640 PMC8223420

[55] Qiu W, Zhang Q, Zhang R, Lu Y, Wang X, Tian H, et al. N6-methyladenosine RNA modification suppresses antiviral innate sensing pathways via reshaping double-stranded RNA. Nat Commun. 2021;12(1):1582. doi: 10.1038/s41467-021-21904-y 33707441 PMC7952553

[56] Wang X, Lu Z, Gomez A, Hon GC, Yue Y, Han D, et al. N6-methyladenosine-dependent regulation of messenger RNA stability. Nature. 2014;505(7481):117–20. doi: 10.1038/nature12730 24284625 PMC3877715

[57] Tsai K, Bogerd HP, Kennedy EM, Emery A, Swanstrom R, Cullen BR. Epitranscriptomic addition of m6A regulates HIV-1 RNA stability and alternative splicing. Genes Dev. 2021;35(13–14):992–1004. doi: 10.1101/gad.348508.121 34140354 PMC8247604

[58] Courtney DG, Kennedy EM, Dumm RE, Bogerd HP, Tsai K, Heaton NS, et al. Epitranscriptomic enhancement of influenza A virus gene expression and replication. Cell Host Microbe. 2017;22(3):377-386.e5. doi: 10.1016/j.chom.2017.08.004 28910636 PMC5615858

[59] Ngwa VM, Edwards DN, Philip M, Chen J. Microenvironmental metabolism regulates antitumor immunity. Cancer Research. 2019;79(16):4003–8. 31362930 10.1158/0008-5472.CAN-19-0617 PMC6697577

[60] Zhang L, Wang Y, Wu G, Xiong W, Gu W, Wang CY. Macrophages: friend or foe in idiopathic pulmonary fibrosis?. Respir Res. 2018;19(1):170. 30189872 10.1186/s12931-018-0864-2 PMC6127991

[61] Liu G, Zhai H, Zhang T, Li S, Li N, Chen J, et al. New therapeutic strategies for IPF: Based on the “phagocytosis-secretion-immunization” network regulation mechanism of pulmonary macrophages. Biomed Pharmacother. 2019;118:109230. doi: 10.1016/j.biopha.2019.109230 31351434

[62] Irizarry-Caro RA, McDaniel MM, Overcast GR, Jain VG, Troutman TD, Pasare C. TLR signaling adapter BCAP regulates inflammatory to reparatory macrophage transition by promoting histone lactylation. Proc Natl Acad Sci U S A. 2020;117(48):30628–38. doi: 10.1073/pnas.2009778117 33199625 PMC7720107

[63] Noe JT, Rendon BE, Geller AE, Conroy LR, Morrissey SM, Young LEA. Lactate supports a metabolic-epigenetic link in macrophage polarization. Sci Adv. 2021;7(46):eabi8602. 10.1126/sciadv.abi8602 PMC8589316 34767443

[64] Kasagi S, Zhang P, Che L, Abbatiello B, Maruyama T, Nakatsukasa H. In vivo-generated antigen-specific regulatory T cells treat autoimmunity without compromising antibacterial immune response. Sci Transl Med. 2014;6(241):241ra78. 10.1126/scitranslmed.3008895 24944193

[65] Gu J, Zhou J, Chen Q, Xu X, Gao J, Li X, et al. Tumor metabolite lactate promotes tumorigenesis by modulating MOESIN lactylation and enhancing TGF-β signaling in regulatory T cells. Cell Rep. 2022;39(12):110986. 35732125 10.1016/j.celrep.2022.110986

[66] Rao K, Zhang X, Luo Y, Xia Q, Jin Y, He J. Lactylation orchestrates ubiquitin-independent degradation of cGAS and promotes tumor growth. Cell Rep. 2025;44(4):115441. doi: 10.1016/j.celrep.2025.115441 40106438

[67] Liao J, Chen Z, Chang R, Yuan T, Li G, Zhu C, et al. CENPA functions as a transcriptional regulator to promote hepatocellular carcinoma progression via cooperating with YY1. Int J Biol Sci. 2023;19(16):5218–32. doi: 10.7150/ijbs.85656 37928273 PMC10620822

[68] Feng F, Wu J, Chi Q, Wang S, Liu W, Yang L, et al. Lactylome analysis unveils lactylation-dependent mechanisms of stemness remodeling in the liver cancer stem cells. Adv Sci (Weinh). 2024;11(38):e2405975. doi: 10.1002/advs.202405975 39099416 PMC11481176

[69] Jin J, Bai L, Wang D, Ding W, Cao Z, Yan P, et al. SIRT3-dependent delactylation of cyclin E2 prevents hepatocellular carcinoma growth. EMBO Rep. 2023;24(5):e56052. doi: 10.15252/embr.202256052 36896611 PMC10157311

[70] Meng Q, Zhang Y, Sun H, Yang X, Hao S, Liu B, et al. Human papillomavirus-16 E6 activates the pentose phosphate pathway to promote cervical cancer cell proliferation by inhibiting G6PD lactylation. Redox Biol. 2024;71:103108. doi: 10.1016/j.redox.2024.103108 38457903 PMC10937312

[71] Yan Q, Zhou J, Gu Y, Huang W, Ruan M, Zhang H, et al. Lactylation of NAT10 promotes N4-acetylcytidine modification on tRNASer-CGA-1-1 to boost oncogenic DNA virus KSHV reactivation. Cell Death Differ. 2024;31(10):1362–74. doi: 10.1038/s41418-024-01327-0 38879723 PMC11445560

[72] Li W, Zhou J, Gu Y, Chen Y, Huang Y, Yang J, et al. Lactylation of RNA m6A demethylase ALKBH5 promotes innate immune response to DNA herpesviruses and mpox virus. Proc Natl Acad Sci U S A. 2024;121(43):e2409132121. doi: 10.1073/pnas.2409132121 39413129 PMC11513906

[73] Tyl MD, Merengwa VU, Cristea IM. Infection-induced lysine lactylation enables herpesvirus immune evasion. Sci Adv. 2025;11(2):eads6215. doi: 10.1126/sciadv.ads6215 39772686 PMC11708889

[74] Murray LA, Sheng X, Cristea IM. Orchestration of protein acetylation as a toggle for cellular defense and virus replication. Nat Commun. 2018;9(1):4967. doi: 10.1038/s41467-018-07179-w 30470744 PMC6251895

[75] Liu B, Tian X, Li L, Zhang R, Wu J, Jiang N, et al. Severe fever with thrombocytopenia syndrome virus induces lactylation of m6A reader protein YTHDF1 to facilitate viral replication. EMBO Rep. 2024;25(12):5599–619. doi: 10.1038/s44319-024-00310-7 39496835 PMC11624280

[76] Pang Y, Zhou Y, Wang Y, Fang L, Xiao S. Lactate-lactylation-HSPA6 axis promotes PRRSV replication by impairing IFN-β production. J Virol. 2024;98(1):e0167023. doi: 10.1128/jvi.01670-23 38088561 PMC10804950

[77] Li H, Yan L, Shi Y, Lv D, Shang J, Bai L, et al. Hepatitis B virus infection: overview. Adv Exp Med Biol. 2020;1179:1–16. doi: 10.1007/978-981-13-9151-4_1 31741331

[78] Gao F, Huang X-L, Cai M-X, Lin M-T, Wang B-F, Wu W, et al. Prognostic value of serum lactate kinetics in critically ill patients with cirrhosis and acute-on-chronic liver failure: a multicenter study. Aging (Albany NY). 2019;11(13):4446–62. doi: 10.18632/aging.102062 31259742 PMC6660055

[79] Nie Y, Liu L-X, Chen T, Zhang Y, Zhu X. Serum lactate level predicts 6-months mortality in patients with hepatitis B virus-related decompensated cirrhosis: a retrospective study. Epidemiol Infect. 2021;149:e26. doi: 10.1017/S0950268820003143 33397544 PMC8057512

[80] Nie Y, Zhang Y, Liu L-X, Zhu X. Serum lactate level predicts short-term and long-term mortality of HBV-ACLF patients: a prospective study. Ther Clin Risk Manag. 2020;16:849–60. doi: 10.2147/TCRM.S272463 32982257 PMC7490053

[81] Chen Y-Y, Wang W-H, Che L, Lan Y, Zhang L-Y, Zhan D-L, et al. BNIP3L-dependent mitophagy promotes HBx-induced cancer stemness of hepatocellular carcinoma cells via glycolysis metabolism reprogramming. Cancers (Basel). 2020;12(3):655. doi: 10.3390/cancers12030655 32168902 PMC7139741

[82] Lamontagne RJ, Casciano JC, Bouchard MJ. A broad investigation of the HBV-mediated changes to primary hepatocyte physiology reveals HBV significantly alters metabolic pathways. Metabolism. 2018;83:50–9. doi: 10.1016/j.metabol.2018.01.007 29410347 PMC5960616

[83] Chen L, Lin X, Lei Y, Xu X, Zhou Q, Chen Y. Aerobic glycolysis enhances HBx-initiated hepatocellular carcinogenesis via NF-κBp65/HK2 signalling. J Exp Clin Cancer Res. 2022;41(1):329. 36411480 10.1186/s13046-022-02531-x PMC9677649

[84] Malik S, Sah R, Muhammad K, Waheed Y. Tracking HPV infection, associated cancer development, and recent treatment efforts–a comprehensive review. Vaccines (Basel). 2023;11(1):102. doi: 10.3390/vaccines11010102 36679945 PMC9860736

[85] Martínez-Ramírez I, Carrillo-García A, Contreras-Paredes A, Ortiz-Sánchez E, Cruz-Gregorio A, Lizano M. Regulation of cellular metabolism by high-risk human papillomaviruses. Int J Mol Sci. 2018;19(7). 10.3390/ijms19071839 PMC6073392 29932118

[86] Martinez-Zapien D, Ruiz FX, Poirson J, Mitschler A, Ramirez J, Forster A, et al. Structure of the E6/E6AP/p53 complex required for HPV-mediated degradation of p53. Nature. 2016;529(7587):541–5. doi: 10.1038/nature16481 26789255 PMC4853763

[87] Jiang P, Liu R, Zheng Y, Liu X, Chang L, Xiong S, et al. MiR-34a inhibits lipopolysaccharide-induced inflammatory response through targeting Notch1 in murine macrophages. Exp Cell Res. 2012;318(10):1175–84. doi: 10.1016/j.yexcr.2012.03.018 22483937

[88] Slabáková E, Culig Z, Remšík J, Souček K. Alternative mechanisms of miR-34a regulation in cancer. Cell Death Dis. 2017;8(10):e3100. doi: 10.1038/cddis.2017.495 29022903 PMC5682661

[89] Zhang R, Su J, Xue S-L, Yang H, Ju L-L, Ji Y, et al. HPV E6/p53 mediated down-regulation of miR-34a inhibits Warburg effect through targeting LDHA in cervical cancer. Am J Cancer Res. 2016;6(2):312–20. 27186405 PMC4859662

[90] Guan P, Howell-Jones R, Li N, Bruni L, de Sanjosé S, Franceschi S, et al. Human papillomavirus types in 115,789 HPV-positive women: a meta-analysis from cervical infection to cancer. Int J Cancer. 2012;131(10):2349–59. doi: 10.1002/ijc.27485 22323075

[91] Arizmendi-Izazaga A, Navarro-Tito N, Jiménez-Wences H, Mendoza-Catalán MA, Martínez-Carrillo DN, Zacapala-Gómez AE. Metabolic reprogramming in cancer: role of HPV 16 variants. Pathogens. 2021;10(3). 10.3390/pathogens10030347 PMC7999907 33809480

[92] Wan N, Wang N, Yu S, Zhang H, Tang S, Wang D, et al. Cyclic immonium ion of lactyllysine reveals widespread lactylation in the human proteome. Nat Methods. 2022;19(7):854–64. doi: 10.1038/s41592-022-01523-1 35761067

[93] Meng Q, Sun H, Zhang Y, Yang X, Hao S, Liu B, et al. Lactylation stabilizes DCBLD1 activating the pentose phosphate pathway to promote cervical cancer progression. J Exp Clin Cancer Res. 2024;43(1):36. doi: 10.1186/s13046-024-02943-x 38291438 PMC10829273

[94] Li S, Bai L, Dong J, Sun R, Lan K. Kaposi’s sarcoma-associated herpesvirus: epidemiology and molecular biology. Adv Exp Med Biol. 2017;1018:91–127. 29052134 10.1007/978-981-10-5765-6_7

[95] Delgado T, Carroll PA, Punjabi AS, Margineantu D, Hockenbery DM, Lagunoff M. Induction of the Warburg effect by Kaposi’s sarcoma herpesvirus is required for the maintenance of latently infected endothelial cells. Proc Natl Acad Sci U S A. 2010;107(23):10696–701. 20498071 10.1073/pnas.1004882107 PMC2890792

[96] Yogev O, Lagos D, Enver T, Boshoff C. Kaposi’s sarcoma herpesvirus microRNAs induce metabolic transformation of infected cells. PLoS Pathog. 2014;10(9):e1004400. doi: 10.1371/journal.ppat.1004400 25255370 PMC4177984

[97] Qi X, Yan Q, Shang Y, Zhao R, Ding X, Gao S-J, et al. A viral interferon regulatory factor degrades RNA-binding protein hnRNP Q1 to enhance aerobic glycolysis via recruiting E3 ubiquitin ligase KLHL3 and decaying GDPD1 mRNA. Cell Death Differ. 2022;29(11):2233–46. doi: 10.1038/s41418-022-01011-1 35538151 PMC9613757

[98] Karki R, Lang SM, Means RE. The MARCH family E3 ubiquitin ligase K5 alters monocyte metabolism and proliferation through receptor tyrosine kinase modulation. PLoS Pathog. 2011;7(4):e1001331. doi: 10.1371/journal.ppat.1001331 21490960 PMC3072377

[99] Yan Q, Zhou J, Wang Z, Ding X, Ma X, Li W. NAT10-dependent N(4)-acetylcytidine modification mediates PAN RNA stability, KSHV reactivation, and IFI16-related inflammasome activation. Nat Commun. 2023;14(1):6327. 37816771 10.1038/s41467-023-42135-3 PMC10564894

[100] Alakunle EF, Okeke MI. Monkeypox virus: a neglected zoonotic pathogen spreads globally. Nat Rev Microbiol. 2022;20(9):507–8. doi: 10.1038/s41579-022-00776-z 35859005 PMC9297671

[101] Weaver JR, Isaacs SN. Monkeypox virus and insights into its immunomodulatory proteins. Immunol Rev. 2008;225:96–113. doi: 10.1111/j.1600-065X.2008.00691.x 18837778 PMC2567051

[102] Moss B. Understanding the biology of monkeypox virus to prevent future outbreaks. Nat Microbiol. 2024;9(6):1408–16. doi: 10.1038/s41564-024-01690-1 38724757

[103] Tyl MD, Betsinger CN, Cristea IM. Virus–host protein interactions as footprints of human cytomegalovirus replication. Curr Opin Virol. 2022;52:135–47. doi: 10.1016/j.coviro.2021.11.016 34923282 PMC8844139

[104] Lum KK, Howard TR, Pan C, Cristea IM. Charge-mediated pyrin oligomerization nucleates antiviral ifi16 sensing of herpesvirus DNA. mBio. 2019;10(4). 10.1128/mBio.01428-19 PMC6650555 31337724

[105] Li T, Diner BA, Chen J, Cristea IM. Acetylation modulates cellular distribution and DNA sensing ability of interferon-inducible protein IFI16. Proc Natl Acad Sci U S A. 2012;109(26):10558–63. doi: 10.1073/pnas.1203447109 22691496 PMC3387042

[106] Zhou C-M, Yu X-J. Unraveling the underlying interaction mechanism between dabie bandavirus and innate immune response. Front Immunol. 2021;12:676861. doi: 10.3389/fimmu.2021.676861 34122440 PMC8190332

[107] Wang Y, Han S, Ran R, Li A, Liu H, Liu M, et al. A longitudinal sampling study of transcriptomic and epigenetic profiles in patients with thrombocytopenia syndrome. Nat Commun. 2021;12(1):5629. doi: 10.1038/s41467-021-25804-z 34561445 PMC8463551

[108] Suga N, Ikeda Y, Yoshikawa S, Taniguchi K, Sawamura H, Matsuda S. In search of a function for the N6-methyladenosine in epitranscriptome, autophagy and neurodegenerative diseases. Neurol Int. 2023;15(3):967–79. doi: 10.3390/neurolint15030062 37606395 PMC10443253

[109] Dokland T. The structural biology of PRRSV. Virus Res. 2010;154(1–2):86–97. doi: 10.1016/j.virusres.2010.07.029 20692304 PMC7114433

[110] Pang Y, Li C, Wang Y, Liu J, Su G, Duan C. Porcine reproductive and respiratory syndrome virus infection manipulates central carbon metabolism. Vet Microbiol. 2023;279:109674. 36739813 10.1016/j.vetmic.2023.109674

[111] Colell A, Green DR, Ricci J-E. Novel roles for GAPDH in cell death and carcinogenesis. Cell Death Differ. 2009;16(12):1573–81. doi: 10.1038/cdd.2009.137 19779498

[112] Liu X, Liu X, Bai J, Gao Y, Song Z, Nauwynck H. Glyceraldehyde-3-phosphate dehydrogenase restricted in cytoplasmic location by viral GP5 facilitates porcine reproductive and respiratory syndrome virus replication via its glycolytic activity. J Virol. 2021;95(18):e0021021. 10.1128/JVI.00210-21 PMC8387053 34160254

[113] Zhang L, Liu X, Mao J, Sun Y, Gao Y, Bai J, et al. Porcine reproductive and respiratory syndrome virus-mediated lactate facilitates virus replication by targeting MAVS. Vet Microbiol. 2023;284:109846. doi: 10.1016/j.vetmic.2023.109846 37586149

[114] Leu J-H, Lin S-J, Huang J-Y, Chen T-C, Lo C-F. A model for apoptotic interaction between white spot syndrome virus and shrimp. Fish Shellfish Immunol. 2013;34(4):1011–7. doi: 10.1016/j.fsi.2012.05.030 22683516

[115] He ST, Lee DY, Tung CY, Li CY, Wang HC. Glutamine metabolism in both the oxidative and reductive directions is triggered in shrimp immune cells (hemocytes) at the WSSV genome replication stage to benefit virus replication. Front Immunol. 2019;10:2102. 31555294 10.3389/fimmu.2019.02102 PMC6737011

[116] Zhang S, Xin F, Zhang X. The compound packaged in virions is the key to trigger host glycolysis machinery for virus life cycle in the cytoplasm. iScience. 2020;24(1):101915. doi: 10.1016/j.isci.2020.101915 33385116 PMC7770649

[117] Xin Q, Wang H, Li Q, Liu S, Qu K, Liu C, et al. Lactylation: a passing fad or the future of posttranslational modification. Inflammation. 2022;45(4):1419–29. doi: 10.1007/s10753-022-01637-w 35224683 PMC9197907

[118] Pan L, Feng F, Wu J, Fan S, Han J, Wang S. Demethylzeylasteral targets lactate by inhibiting histone lactylation to suppress the tumorigenicity of liver cancer stem cells. Pharmacol Res. 2022;181:106270. 35605812 10.1016/j.phrs.2022.106270

[119] Sharma D, Singh M, Rani R. Role of LDH in tumor glycolysis: regulation of LDHA by small molecules for cancer therapeutics. Semin Cancer Biol. 2022;87:184–95. doi: 10.1016/j.semcancer.2022.11.007 36371026

[120] Zhou L, He R, Fang P, Li M, Yu H, Wang Q, et al. Hepatitis B virus rigs the cellular metabolome to avoid innate immune recognition. Nat Commun. 2021;12(1):98. doi: 10.1038/s41467-020-20316-8 33397935 PMC7782485

[121] Li H, Lin C, Qi W, Sun Z, Xie Z, Jia W, et al. Senecavirus A-induced glycolysis facilitates virus replication by promoting lactate production that attenuates the interaction between MAVS and RIG-I. PLoS Pathog. 2023;19(5):e1011371. doi: 10.1371/journal.ppat.1011371 37126525 PMC10174517

[122] Xing B-C, Wang C, Ji F-J, Zhang X-B. Synergistically suppressive effects on colorectal cancer cells by combination of mTOR inhibitor and glycolysis inhibitor, oxamate. Int J Clin Exp Pathol. 2018;11(9):4439–45. 31949841 PMC6962984

[123] Sun S, Li H, Chen J, Qian Q. Lactic acid: no longer an inert and end-product of glycolysis. Physiology (Bethesda). 2017;32(6):453–63. doi: 10.1152/physiol.00016.2017 29021365

[124] Beloueche-Babari M, Casals Galobart T, Delgado-Goni T, Wantuch S, Parkes HG, Tandy D, et al. Monocarboxylate transporter 1 blockade with AZD3965 inhibits lipid biosynthesis and increases tumour immune cell infiltration. Br J Cancer. 2020;122(6):895–903. doi: 10.1038/s41416-019-0717-x 31937921 PMC7078321

[125] D’Aria S, Maquet C, Li S, Dhup S, Lepez A, Kohler A, et al. Expression of the monocarboxylate transporter MCT1 is required for virus-specific mouse CD8^+^ T cell memory development. Proc Natl Acad Sci U S A. 2024;121(13):e2306763121. doi: 10.1073/pnas.2306763121 38498711 PMC10990098

[126] Lv X, Lv Y, Dai X. Lactate, histone lactylation and cancer hallmarks. Expert Rev Mol Med. 2023;25:e7. 10.1017/erm.2022.42 36621008

[127] Li W, Zhou C, Yu L, Hou Z, Liu H, Kong L, et al. Tumor-derived lactate promotes resistance to bevacizumab treatment by facilitating autophagy enhancer protein RUBCNL expression through histone H3 lysine 18 lactylation (H3K18la) in colorectal cancer. Autophagy. 2024;20(1):114–30. doi: 10.1080/15548627.2023.2249762 37615625 PMC10761097

[128] Deng T, Du L, Ding S, Peng X, Chen W, Yan Y, et al. Protein kinase Cdc7 supports viral replication by phosphorylating avibirnavirus VP3 protein. J Virol. 2023;97(11):e0112523. doi: 10.1128/jvi.01125-23 37902398 PMC10688373

[129] Kostyusheva A, Brezgin S, Glebe D, Kostyushev D, Chulanov V. Host-cell interactions in HBV infection and pathogenesis: the emerging role of m6A modification. Emerg Microbes Infect. 2021;10(1):2264–75. doi: 10.1080/22221751.2021.2006580 34767497 PMC8648018

[130] He Y, Song T, Ning J, Wang Z, Yin Z, Jiang P, et al. Lactylation in cancer: mechanisms in tumour biology and therapeutic potentials. Clin Transl Med. 2024;14(11):e70070. doi: 10.1002/ctm2.70070 39456119 PMC11511673

[131] Huang T, Feng Q, Wang Z, Li W, Sun Z, Wilhelm J, et al. Tumor-targeted inhibition of monocarboxylate transporter 1 improves T-cell immunotherapy of solid tumors. Adv Healthc Mater. 2021;10(4):e2000549. doi: 10.1002/adhm.202000549 32431046 PMC7674253

[132] Su J, Zheng Z, Bian C, Chang S, Bao J, Yu H, et al. Functions and mechanisms of lactylation in carcinogenesis and immunosuppression. Front Immunol. 2023;14:1253064. doi: 10.3389/fimmu.2023.1253064 37646027 PMC10461103

